# Synthesis and Spectroscopic Investigations of Schiff Base Ligand and Its Bimetallic Ag(I) Complex as DNA and BSA Binders

**DOI:** 10.3390/biom11101449

**Published:** 2021-10-02

**Authors:** Martyna Szymańska, Izabela Pospieszna-Markiewicz, Martyna Mańka, Małgorzata Insińska-Rak, Grzegorz Dutkiewicz, Violetta Patroniak, Marta A. Fik-Jaskółka

**Affiliations:** 1 Faculty of Chemistry, Adam Mickiewicz University in Poznań, Uniwersytetu Poznańskiego 8, 61-614 Poznań, Poland; martyna.szymanska@amu.edu.pl (M.S.); izap@amu.edu.pl (I.P.-M.); marman9@st.amu.edu.pl (M.M.); inka@amu.edu.pl (M.I.-R.); gdutkiew@amu.edu.pl (G.D.); violapat@amu.edu.pl (V.P.); 2 Centre for Advanced Technology, Adam Mickiewicz University in Poznań, Uniwersytetu Poznańskiego 10, 61-614 Poznań, Poland

**Keywords:** Schiff base, silver(I) complex, BSA, CT-DNA, anticancer, anti-inflammatory

## Abstract

Generation of well-defined potential metallotherapeutics for cancer treatment, one of the most population-threatening diseases, is challenging and an active area of modern research in view of their unique properties and thus multiple possible pathways of action in cells. Specifically, Schiff base ligands were recognized as very promising building blocks for the construction of stable and active complexes of numerous geometries and topologies. Incorporation of Ag(I) ions allows for the formation of flat complexes with potential unoccupied coordination sites, thus giving rise to specific interactions between the metallotherapeutic and biomolecule of interest. Herein, we present the design, synthesis and characterization of new Schiff base ligand **L** and its Ag(I) bimetallic complex **[Ag_2_L_2_]^2+^** with two planar moieties formed around the metal ions and connected through cyclohexane rings, confirmed by X-ray measurements. The compounds were described in context of their potential use as anticancer drugs through DNA and BSA binding pathways by several spectroscopic methods (CD, UV-Vis, fluorescence). We revealed that both, **L** and **[Ag_2_L_2_]^2+^**, interact with similar affinity with CT-DNA (K_b_~10^6^ M^−1^), while they differ in the type and strength of interactions with the model albumin–BSA. **[Ag_2_L_2_]^2+^** binds BSA in both a dynamic and static manner with the K_sv_ = 8.8 × 10^4^ M^−1^ in the Trp-134 and Trp-213 sites, whereas **L** interacts with BSA only dynamically (K_SV_ = 2.4 × 10^4^ M^−1^). This found further confirmation in the CD studies which revealed a reduction in α-helix content in the albumin of 16% in presence of **[Ag_2_L_2_]^2+^**.

## 1. Introduction

The antimicrobial properties of silver ions have been known since ancient times [1]. They are toxic to certain types of bacteria, viruses and fungi, however, their toxicity to mammalian cells is low, enabling the use of silver compounds in cancer therapies [2]. This metal has been found in trace amounts in 29 human tissues, but the importance of silver in the functioning of the body has never been established. Despite the lack of toxicity, absorption of too much silver results in argyria, a permanent hyperpigmentation leading to a steel gray tint of the skin [3,4].

For many years, silver compounds have been studied for their biological activity as potential drugs for chronic skin ulcers, and open and festering wounds [5]. However, with the introduction of cisplatin as a commonly used chemotherapeutic agent, the interest of researchers from all over the world has focused on the invention of other metallotherapeutics [2]. The disadvantage of *cis*platin and its derivatives (carboplatin and oxaliplatin) are extended toxicity and resistance of tumors, therefore finding an alternative, e.g., in the form of silver compounds, could be a milestone in cancer therapy [6].

When designing silver coordination compounds, special attention should be paid to factors such as the strength of the bond between the ligand and the silver ion [7], ability of the silver ion to reach a coordination number from two to six, selection of appropriate ligands with specific electronic, spherical and steric features, at the same time containing donor atoms such as nitrogen, phosphorus and sulfur as well as amino acids and carboxylic acids [8], and also functional groups, while controlling the issues of stability of the complexes, silver release, solubility of the complex in water or fats and redox properties ^2^. For example, the Ag(I) saccharinate complex has been described as showing potential in the treatment of human lung carcinoma and human breast adenocarcinoma [9]. Similarly promising in the context of anticancer activity are coordination compounds of Ag(I) with ligands based on carbenes, phosphines, polypyridines and coumarins [10,11,12,13,14]. Moreover, recent studies aim to investigate the Schiff base systems as potential biomedical agents. The elegant and efficient syntheses, together with high moisture and thermal stability [15,16] in physiological pH of Schiff bases and their metal complexes, became the foundations for the research on their antibacterial, antimalarial, antiproliferative and antioxidant properties [17,18,19,20,21,22].

The problems limiting the applicability of silver complexes are poor aqueous solubility and lack of stability to light [8]. However, at the same time, due to the photosensitivity of Ag(I) compounds, research is being carried out on their use as photoactive drugs. This would allow activation of the chemotherapeutic only after irradiation at the target site, ensuring control of the drug and thus reducing the side effects that occur with standard chemotherapy [23]. According to the Pearson classification, Ag(I) belongs to the soft cations [24]. This means that it binds to soft polarizable ligands. Silver, thanks to the back donation of electrons from its d orbitals to the π* orbitals of the ligand, takes part in the formation of strong σ − π bonds [25].

The interaction of metal ions with proteins or nucleic acids plays a very important role in biological processes necessary for the proper functioning of the body. It enables the transport and storage of metals, their complexes and the transfer of electrons. Some metal complexes containing labile ligands are used as drugs whose action is based on the exchange of the ligand with the target enzyme [26,27]. The action of Ag(I) complexes involves the slow release of silver ions, which can then interact with DNA and proteins or lead to the production of reactive oxygen species, resulting in cell death [28].

There are several possibilities for interactions of metal complex compounds with DNA. These include covalent and non-covalent bonds. Covalent bonding involves the exchange of a labile ligand that is part of a coordination compound for one of the DNA bases [29] in which the N7 atoms of adenine and guanine are preferred [30] which affects the electron density of the heterocycles, causing instability of the DNA helix. The large number of binding sites disrupt the hydrogen bonding and stacking of nitrogenous bases [31]. Silver complexes can also bind to DNA through electrostatic binding to the phosphate backbone or binding in a major or minor groove of helix [32,33,34,35]. It has been shown that adenine- and thymine-rich regions, due to the fact that they form narrower and better-fitting pockets than cytosine- and guanine-rich sequences, are more suitable for docking small flexible complex molecules, whereas pockets formed by cytosine- and guanine-rich regions are preferred by complex molecules with a large size and rigid core [23,29]. However, when interacting with DNA, silver ions choose nitrogen bases rather than the phosphate backbone [1]. The binding of silver complexes to DNA results in the unfolding of a region with condensed DNA, leading to a halt in replication [36]. This leads to disruption of genomic and cellular processes, which affects changes in the regulation of DNA transcription [37,38], as well as cell apoptosis. The induction of programmed cell death is a well-known action of chemotherapeutic agents, but interactions with RNA, certain enzymes and the mitochondria are also possible [29]. Silver coordination compounds can also intercalate between DNA nitrogen base pairs, thereby causing the helix to distort, unfold and lengthen [23,39]. It has been proven that the selectivity of silver coordination compounds towards cell lines is related to the stability of the complexes and the hydrophilic–lipophilic balance, which is greatly influenced by the type of ligand used [40].

Silver ions can attach to amino acids in three different ways: by coordination to the donor nitrogen amine atom at the N-terminus (amino acid as a bidentate ligand), by coordination to the oxygen atom making up the carboxyl group at the C-terminus (possible interaction with both oxygen atoms) and by coordination to the heteroatoms included in the side chains (amino acid as a bidentate or tridentate ligand) [27,41]. According to the hybrid density functional theory, silver ions should bind most strongly to arginine, lysine and histidine and least strongly to non-polar aliphatic amino acids (with the exception of methionine) [42]. However, it is believed that cysteine and methionine are the preferential binding sites for Ag(I) due to the presence of sulfur atom (the softest group in proteins) [43]. For example, interaction of Ag(I) ion with the thiol group of the L-cysteine residue of proteins results in inhibition of enzyme activity, their neutralization and formation of reactive oxygen species [1,36]. The Ag(I) ion also strongly binds to other anionic protein ligands [44].

Therefore, herein, we aimed to design and synthesize a new potential drug able to transport to the cancerous and inflammatory cells through binding with serum albumins and to act as a DNA binder, leading to apoptosis of harmful cells. The potency of Schiff base ligand **L** and its **[Ag_2_L_2_]^2+^** complex as drugs was evaluated by spectroscopic techniques—CD, UV-Vis, fluorescence and synchronous fluorescence. This versatile approach aiming to selectively eliminate the unwanted cell may be the key for future targeted therapies.

## 2. Materials and Methods

### 2.1. Materials and Methods

CT-DNA, BSA, ethidium bromide, Hoechst 33258, Tris, PBS, AgPF_6_ and NaCl were supplied from Sigma-Aldrich and used without further purification. ESI mass spectra for MeCN solutions ~10^−4^ M were measured using a Waters Micromass ZQ spectrometer and QTOF type mass spectrometer Impact HD, Bruker. NMR spectra were run on a Varian Gemini 400 MHz spectrometer and were calibrated against the residual protonated solvent signals with chemical shifts represented in ppm. Microanalyses were performed using VarioEL III CHN element analyzer. The FT-IR spectra were recorded in a range between 4000 and 400 cm^−1^ using Bruker FT-IR IFS 66/s spectrometer. The potential toxicity class of **L**, **[Ag_2_L_2_](PF_6_)_2_** and *cis*platin (as a reference) were evaluated on the ProTox web based server (tox.charite.de). The toxicities are classified in classes I–VI according to the GHS United Nations 2015 [45].

CT-DNA was dissolved in Tris Buffer (5 mM Tris HCl, 50 mM NaCl, pH = 7.2) prior to use. The CT-DNA solution gave a ratio of UV absorbance of 1.82:1 at 260 and 280 nm, indicating that the CT-DNA sample was sufficiently free from protein [46,47]. CT-DNA concentration per nucleotide was determined from the UV absorbance at 260 nm using the extinction coefficient ε_260_ = 6600 dm^3^·mol^−1^·cm^−1^ [48]. d(GTTAATCGCTGG) was dissolved in (0.5 mM (CH_3_)_2_AsO_2_H, 0.05 mM NaOH, pH = 7.2) and subjected to renaturation. BSA solution was prepared at a 75 nM concentration based on its molecular weight (MW66 kDa) using PBS (137 mM NaCl, 2.7 mM KCl, 10 mM Na_2_HPO_4_, 1.8 mM KH_2_PO_4_, pH =7.4). Additionally, BSA solution was checked spectroscopically and stored in the dark at 4 °C before measurements. Electronic absorption spectra were performed on UV–Vis JASCO V-770 equipped with a Peltier Thermo Cell Holder (water) PAC-743R. Emission spectra in the competitive fluorescence titration experiments were measured at room temperature on an Agilent Technologies Cary Eclipse Fluorescence Spectrophotometer G9800A. CD spectra of BSA were obtained on a J-810 spectropolarimeter (Jasco Europe S.R.L., Cremella, Italy) equipped with a Peltier PTC-423S/15. Synchronous fluorescence and fluorescence quenching spectroscopic studies of BSA were carried out on spectrofluorimeter Fluorolog 3–22 (Horiba-Jobin_Yvon). For all spectral measurements, 10 × 10 mm quartz cells were used. Stock solutions of compounds in acetonitrile (at concentration 2 × 10^−3^ M) were prepared by dissolving of crystals and were taken for all spectroscopic experiments. It needs to be emphasized that compounds are stable in this medium for several days (after this time some precipitate starts to occur). The stability of **L** and **[Ag_2_L_2_](PF_6_)_2_** in 1% solution of MeCN in Tris-HCl buffer (5 mM Tris HCl, 50 mM NaCl, pH = 7.2) and PBS buffer (137 mM NaCl, 2.7 mM KCl, 10 mM Na_2_HPO_4_, 1.8 mM KH_2_PO_4_, pH = 7.4) was confirmed within 120 min.

### 2.2. Synthesis of Ligand L

**Scheme 1:**
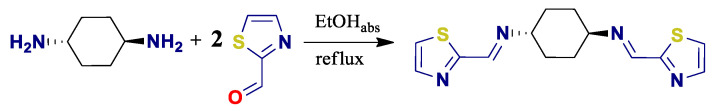
Synthesis of ligand **L**.

The ligand was synthesized according to the procedure depicted in Scheme 1.

The 1,4-trans-diaminocyclohexane (107.12 mg, 0.94 mmol) was dissolved in 5 mL absolute EtOH. Then 2-thiazolecarboxaldehyde (212.27 mg, 1.88 mmol, 164.8 μL) was added. The reaction was carried out for 24 h under an inert atmosphere at 80 °C. The product was filtered under reduced pressure, washed with cold absolute EtOH and dried under reduced pressure for 5 h. A yellow product was obtained in 72.0% yield.

^1^H NMR (400 MHz, CD_3_CN-d_3_): δ(ppm) = 8.51 (s, 1H); 7.90 (d, 1H); 7.55 (d, 1H); 3.47 (m, 1H); 1.83 (m, 2H); 1.71 (m, 2H).
^13^C NMR (75 MHz, DMSO-d_6_): δ(ppm) = 166.78; 153.66; 144.04; 122.64; 67.04; 31.69 (2C).
ESI-MS(+) m/z(%): 305 (30) [**L**+H]^+^; 327 (10) [**L**+Na]^+^.
Anal. calcd. for (C_14_H_16_N_4_S_2_): C: 55.23, H: 5.30, N: 18.40, S: 21.07%; found: C: 55.13, H: 5.34, N: 18.48, S: 21.05%.
FT-IR (KBr): 3446, ν(O−H); 2929, 2854 ν (C−H)_aliph_; 1640 ν(C=N); 1486 ν(C=C); 1447, 1420 δ(C-H); 1376, 1319 ν(C−N); 1223, 1147 ν(C−S); 947, 893, 780, 699 ν(C-H)_arom_ cm^−1^.

### 2.3. Synthesis of Complex [Ag_2_L_2_](PF_6_)_2_

The ligand **L** (20.4 mg, 0.067 mmol) was dissolved in 10 mL acetonitrile. After heating, the solution was clear and had a yellow color. AgPF_6_ salt (16.94 mg, 0.067 mmol) was then added to the solution. The solution still turned yellow. The flask was wrapped in aluminum foil due to the silver compounds being photosensitive and the reaction was stirred for 24 h. The solution was concentrated on an evaporator to a minimum amount. Precipitate began to fall out, then was filtered and dried under reduced pressure for at least 5 h. Transparent yellow monocrystals suitable for X-ray analysis were obtained by slow diffusion of diethyl ether into acetonitrile solution of the complex at 4 °C. Yield: 47.5%

^1^H NMR (400 MHz, CD_3_CN–d_3_): δ(ppm) = 8.58 (s, 1H); 7.97 (d, 1H); 7.77 (d, 1H); 3.48 (m, 1H); 1.91 (m, 2H); 1.69 (m, 2H).
ESI-MS(+) m/z(%): 412.98 (100) **[AgL]^+^**; 717.07 (16) **[AgL_2_]^+^**; 968.93 (2) {**[Ag_2_L_2_]**(PF_6_)}^+^.
Anal. calcd. for (C_28_H_32_Ag_2_N_8_S_4_P_2_F_12_): C: 30.17, H: 2.89, N: 10.05, S: 11.51%; found: C: 30.62, H: 2.72, N: 10.34, S: 11.91%.
FT-IR (KBr): 3461 ν(O−H); 2930, 2858 ν(C−H)_aliph_; 1641, 1629 ν(C=N); 1485 ν(C=C); 1382 δ(C-H); 1323 ν(C−N); 1206, 1136 ν(C−S); 1075, 960, 628 ν(C-H)_arom_; 840 PF_6_^-^; 559 ν(Ag−N) cm^−1^.

### 2.4. X-ray Crystallography

X-ray diffraction data were collected by the ω-scan technique, for (Ag) at 100(1) K on Rigaku four-circle Xcalibur diffractometer (Eos detector) with graphite-monochromatized MoK_α_ radiation (*λ* = 0.71073) Å, and for (L) at room temperature on Rigaku four-circle Supernova diffractometer (Atlas detector) with mirror-monochromatized CuK_α_ radiation (*λ* = 1.54184) Å. The data were corrected for Lorentz–polarization and absorption effects [49]. The structures were solved with SHELXT [50] and refined with the full-matrix least-squares procedure on F^2^ by SHELXL [51]. All non-hydrogen atoms were refined anisotropically, hydrogen atoms were placed in the calculated positions and refined as ‘riding model’ with the isotropic displacement parameters set at 1.2 times the U_eq_ value for appropriate non-hydrogen atom.

Crystal data: **(L)**: C_14_H_16_N_4_S_2_, M_r_ =304.43, monoclinic, P2_1_/n, a = 6.3822(2) Å, b = 7.15988(2) Å, c = 17.0967(4) Å, β = 97.276(2)°, V = 774.95(3) Å^3^, Z = 2, T = 291 K, d_x_ = 1.305 g/cm^3^, F(000) = 320, μ = 3.071 mm^−1^, 3035 reflection collected, 1566 symmetry independent (R_int_ = 2.52%), 1432 with I>2σ(I). Final R[I > 2σ(I)] = 0.0392, wR2[I > 2σ(I)] = 0.1086, R[all reflections] = 0.0428, wR2 [all reflections] = 0.1164, S = 1.076, (Δρ_max_/ Δρ_min_) = 0.31/−0.25 e·Å^−3^.

**([Ag_2_L_2_]^2+^)**: (C_28_H_32_Ag_2_N_8_S_4_)^2+^·2(PF_6_)^-^·2(CH_3_CN), M_r_ = 1196.64, monoclinic, P2_1_/n, a = 12.0006(4) Å, b = 10.1569(3) Å, c = 18.0573(7) Å, β = 97.693(3)°, V = 2181.17(13) Å^3^, Z = 2, d_x_ = 1.82 g·cm^−3^, F(000) = 1192, μ = 1.253 mm^−1^, 8929 reflection collected, 4376 symmetry independent (R_int_ = 2.64%), 3671 with I>2σ(I). Final R[I>2σ(I)] = 0.0367, wR2[I>2σ(I)] = 0.0817, R [all reflections] = 0.0470, wR2 [all reflections] = 0.0881, S = 1.034, (Δρ_max_/ Δρ_min_) = 0.97/−0.86 e·Å^−3^.

Crystallographic data for the structural analysis have been deposited with the Cambridge Crystallographic Data Centre, Nos. CCDC–2104856 (**L**) and CCDC–2094959 (**[Ag_2_L_2_]^2+^**). Copies of this information may be obtained free of charge from: The Director, CCDC, 12 Union Road, Cambridge, CB2 1EZ, UK. Fax: +44(1223)336-033, e-mail: deposit@ccdc.cam.ac.uk, or www: www.ccdc.cam.ac.uk (accessed on 30 September 2021).

### 2.5. DNA Binding Studies

#### 2.5.1. UV-Vis Absorption Titration

Increasing amounts of CT-DNA (0-100 μM) were added to the solutions of the ligand and complex (20 μM) in Tris buffer (5 mM Tris HCl, 50 mM NaCl, pH = 7.2). Time intervals of 5 min were maintained before each measurement. The measurements were performed in the range of 800–200 nm. The binding constants were evaluated using a modern non-linear regression fitting method for stoichiometry 1:1 from the free online BindFit v0.5 module (http://www.supramolecular.org (accessed on 30 September 2021)). UV fitting was performed using the Nelder–Mead method by taking the absorbance values at both wavelengths of 290 and 231 nm [52,53,54].

#### 2.5.2. Fluorescence Competitive Binding with Ethidium Bromide

Ethidium bromide (EB, 20 μM) and CT-DNA (26 μM) were added to Tris buffer (5 mM Tris HCl, 50 mM NaCl, pH = 7.2), then the solution was incubated in darkness for 30 min. After this time, fluorescence measurements were performed. Later, portions of stock solution of ligand or complex were added to sample in the concentration range 0-190 μM). Before each measurement the samples were equilibrated for 5 min. The tests were carried out in the range of 720–540 nm at λ_exc_ = 467 nm.

#### 2.5.3. Fluorescence Competitive Binding with Hoechst33258

The solution of Hoechst (10 µM) with CT-DNA (20 µM) in 5mM Tris HCl 50 mM NaCl buffer (pH = 7.2) were allowed to equilibrate for 30 min at 25 °C in darkness before the titrations were performed. The emission spectra of Hoechst-DNA with increasing concentration of the complex or ligand (0–150 µM) were recorded in the range 650–400 nm at λ_exc_ = 363 nm.

#### 2.5.4. UV Melting Experiments

Next, 10 μM solutions of complex **[Ag_2_L_2_]^2+^** or ligand **L** were added to the quartz cuvettes and the baselines were measured. Then the double-stranded DNA was added to the cuvettes to achieve a 2.5 μM concentration. Measurements were performed recording the absorbance at a 260 nm wavelength at a rate of 1 °C/min, within the 10–95 °C temperature range. Melting temperature (T_m_) values were determined as the temperatures relative to maxima of the 1st derivative plots of denaturation curves. Presented curves are averages of at least two experiments. Sodium cacodyl buffer (0.5 mM (CH_3_)_2_AsO_2_H, 0.05 mM NaOH, pH = 7.2) was used in the measurements.

### 2.6. Protein Binding Studies

#### 2.6.1. CD Analysis

To the solution of BSA (75 nM) in PBS buffer (137 mM NaCl, 2.7 mM KCl, 10 mM Na_2_HPO_4_, 1.8 mM KH_2_PO_4_, pH = 7.4) the increasing amounts of solutions of **L** (0–26.25 μM) or **[Ag_2_L_2_](PF_6_)_2_** (0–26.25 μM) were added. All spectra were measured in the 260–201 nm range. The means of residue ellipticity were calculated from the equation below [55]:

(1) $$MRE=\frac{ObservedCD\;\left[mdeg\right]}{C_{p}nl\times 10},\;[\frac{\deg\times {\mathrm{cm}}^{2}}{\mathrm{dmol}}],$$

where n is number of amino acid residues (n is 582 for BSA), C_p_ is the molar concentration of the protein [$\frac{\mathrm{mol}}{{\mathrm{dm}}^{3}}]$, l is the optical path [cm].

The α-helical content of free and bound BSA was obtained using the formula [55]:

(2) $$\alpha -\mathrm{Helical}(\%)=\frac{-MRE_{208}-4000}{33000-4000}\times 100,$$

where 330,00 is the value of the MRE for pure α-helix at 208 nm and 4000 is the MRE value at 208 nm for random coil conformation and β-form cross.

#### 2.6.2. Fluorescence Quenching Studies

The 5 μM solution of BSA in PBS buffer (137 mM NaCl, 2.7 mM KCl, 10 mM Na_2_HPO_4_, 1.8 mM KH_2_PO_4_, pH = 7.4) was titrated with the solutions of **L** (0–170 μM) or **[Ag_2_L_2_](PF_6_)_2_** (0–110 μM). Measurements were carried out with 2/2 slits at λ_exc_ = 292 nm in the range 570–300 nm. The quenching constants were calculated on the basis of the equation [56]:

I_0_/I = 1 + K_SV_[Q], (3)

where I and I_0_ are fluorescence intensities in presence and absence of the quencher, K_SV_ is the Stern–Volmer constant and [Q] is concentration of the quencher.

#### 2.6.3. Number of Binding Sites and Binding Constant

The Scatchard equation was used for calculation of the static quenching parameters which are the binding constant K_bin_ and the number of binding sites n [57]:

(4) $$\log\left[\frac{F_{0}-F}{F}\right]=\log K_{b}+n\log\left[Q\right],$$

where [Q] is concentration of a quencher, n is number of binding sites, F_0_ and F are fluorescence without and with quencher, and K_b_ is the binding constant of a quencher established from the slope of $\log\left[\frac{F_{0}-F}{F}\right]$ compared to log[Q].

#### 2.6.4. Synchronous Fluorescence Studies

The 5 μM solution of BSA in PBS buffer (137 mM NaCl, 2.7 mM KCl, 10 mM Na_2_HPO_4_, 1.8 mM KH_2_PO_4_, pH = 7.4) was titrated with increasing concentrations of **L** or **[Ag_2_L_2_](PF_6_)_2_** (in the concentration range 0–70 μM). Measurements were carried out with excitation wavelength λ_exc_ = 292 nm in the range of 400–250 nm, with 2/2 slits and 15 or 60 nm offsets for Tyr and Trp, respectively.

## 3. Results and Discussion

### 3.1. Design, Synthesis and Characterization

**Scheme 2:**
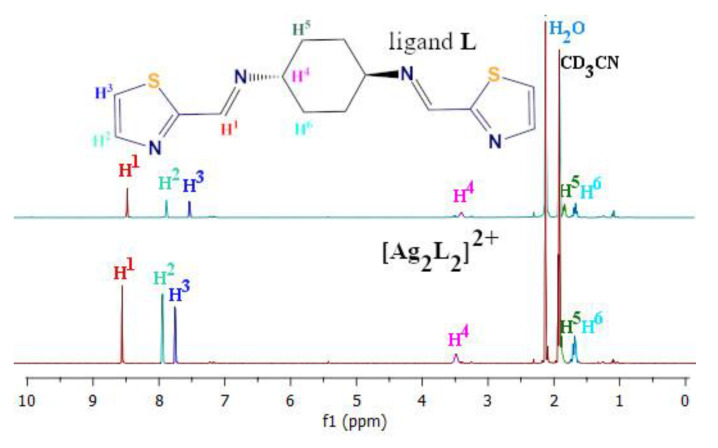
NMR spectra of **L** and **[Ag_2_L_2_]^2+^** in acetonitrile-d_3_ (400 MHz, 298 K).

Schiff bases are particularly potent ligands due to their feasible preparation and diversity. A smart selection of amines and aldehydes or ketones allows us to easily obtain ligands with desired active sites and proper coordination moieties [58]. Since thiazole has attracted lots of interest over the years due to its numerous pharmacological applications, it has been included in our study [59]. Herein, the N_4_-donor Schiff base ligand **L** is a product of condensation of one equiv. of 1,4-*trans*-diaminocyclohexane and two equiv. of 2-thiazolecarboxaldehyde. The ProTox online tool [60] has classified **L** as toxic (toxicity class III) similarly to *cis*platin, an anticancer drug with numerous side effects. Interestingly, coordination of Ag(I) reduced the predicted toxicity to class IV (harmful), leading, potentially, to less severe side effects during therapy. Therefore, the reaction of ligand **L** and silver(I) hexafluorophosphate was carried out in a 1:1 molar ratio in ethanol and led to a bimetallic complex **[Ag_2_L_2_]^2+^** with two planar moieties formed by thiazoles around the metal ions and connected by cyclohexane rings. Interestingly, according to X-ray diffraction of a single crystal, the ligand **L** with the potentially tetradentate set of donor atoms acts as a tridentate one (*cf.* 3.2). The coordination of Ag(I) was also confirmed by the IR spectra (Figure S1). The strong band at 1640 cm^−1^ in the free ligand, attributable to (C=N), confirms formation of Schiff base [61]. In complex this bond is visible as two peaks at 1629 and 1641 cm^−1^ suggesting that only one imine moiety takes part in coordination. The coordination is also supported by the occurrence of the bands at 559 cm^−1^ arising from the Ag-N bonding and at 837 cm^−1^ arising from the presence of PF_6_^-^ anions. The in-solution structures of **L** and **[Ag_2_L_2_]^2+^** were determined by the NMR spectra in acetonitrile (Scheme 2, Figure S2). In **[Ag_2_L_2_]^2+^** the H1 and H2 are slightly (*ca.* 0.07 ppm) shifted downfield, while H3 is shifted 0.22 ppm with regards to the spectrum of **L**. The shift of H3 signal corresponds to the coordination of Ag(I) by N in thiazole as may be concluded from the resonance structure of this five-membered heterocycle [62]. Interestingly, only one signal attributed to the imine (CH=N) protons (H1) is visible suggesting that Ag(I) ions are bi- or tetracoordinated in solution. ESI-MS confirms formation of the ligand with the peaks at m/z = 305 [H**L**]^+^ and 327 [Na**L**]^+^, while the presence of **[Ag_2_L_2_](PF_6_)_2_** is confirmed by the peak at m/z = 968.93 {**[Ag_2_L_2_]**(PF_6_)]}^+^ The stability of the compounds was also confirmed by UV in a 1% solution of acetonitrile in buffers (5 mM Tris HCl, 50 mM NaCl, pH = 7.2) and (137 mM NaCl, 2.7 mM KCl, 10 mM Na_2_HPO_4_, 1.8 mM KH_2_PO_4_, pH = 7.4), the basic media used in the spectroscopic studies on the interactions with biomolecules (Figures S3 and S4).

### 3.2. X-ray Structures

**Figure 1:**
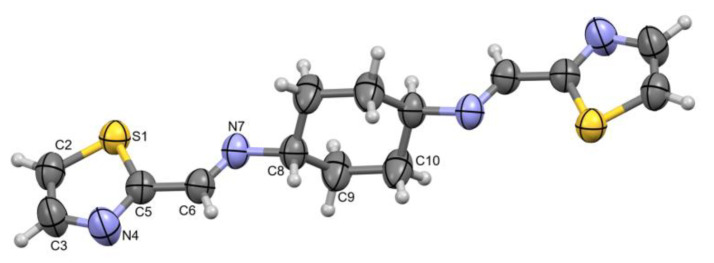
A perspective view of the ligand molecule as it exists in the crystal structure. The ellipsoids are drawn at the 50% probability level, hydrogen atoms are represented by the spheres of arbitrary radii. The unlabeled part of the ligand is related to the labeled one by the symmetry operation 1-x,2-y,1-z.

**Figure 2:**
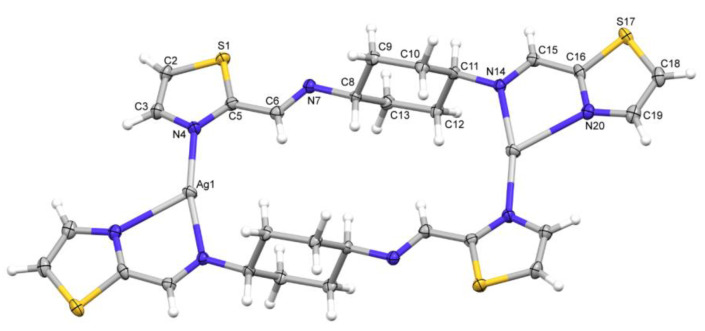
A perspective view of the dicationic complex as it exists in the crystal structure. The ellipsoids are drawn at the 50% probability level; hydrogen atoms are represented by the spheres of arbitrary radii. The unlabeled part of the complex is related to the labeled one by the symmetry operation 1-x,2-y,1-z.

Figure 1 and Figure 2 show the perspective views of the ligand and the complex, respectively, together with the numbering schemes. In the crystal structure ligand (**L**) is centrosymmetric (*C_i_*); as a consequence, the planes of the five-membered rings are exactly parallel. Incidentally, the two-centered **[Ag_2_L_2_]^2+^** complex in the crystal structure is also *C_i_*-symmetrical, as it also lies across the inversion center. The asymmetric part contains therefore one half of the dicationic complex, one PF_6_^-^ anion, and one solvent–acetonitrile molecule (Figure S5). The silver centers are coordinated by three nitrogen atoms (two from one ligand molecule and one from the other), in highly distorted triangular fashion. The Ag ion lies roughly in the plane of the N_3_ coordination group; the deviation from this plane is 0.052 Å (Table 1).

### 3.3. Interactions with DNA

DNA is one of the main targets of many metallodrugs currently used in clinical treatments and trials. This is due to the vast amount of detailed structural information available and the richness of possible binding motifs [63]. Since several mechanisms may lead a synthetic ligand to bind to nucleic acid structure [64], it is clearly necessary to investigate the nature of molecular recognition, especially in the evaluation of DNA-binding ability of new compounds.

In general, compounds may interact with DNA helix via several different modes. In terms of specific interactions, the most relevant possibilities arise from the individual shape, charge and flexibility of the compounds. Components possessing flat moieties may slide between the nucleobases, leading to the expansion of the helix and distortion of its structure. Intercalators, such as EB and doxorubicin, strongly bind with DNA and often lead to severe side effects on the healthy cells [65]. More bulky compounds, such as supramolecular helicates, may bind in the minor or major grooves of the DNA. This kind of interaction may be supported by van der Waals and specific hydrophobic interactions with aromatic rings of nucleobases, such as π-π T-shaped, π-alkyl or π-hydrogen bond donor [13,66].

Spectroscopic methods, including electronic absorption titration [67] and fluorescent competitive binding studies with EB [68], can furnish important details concerning the type of interactions between potential drugs and the DNA and were employed thus in the present study. Hence, DNA binding of our newly obtained compounds was characterized, in a comparative manner as discussed below.

#### 3.3.1. Electronic Absorption Titration

**Figure 3:**
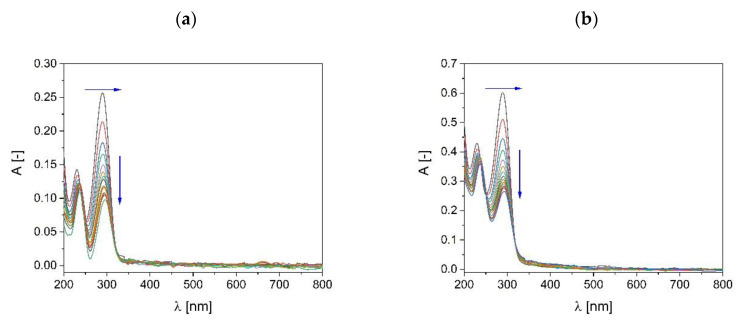
Absorption spectra of (**a**) **L** and (**b**) **[Ag_2_L_2_]^2+^** in the presence of increasing concentrations of CT-DNA (0–100 μM); ([compound] = 20 μM). Arrows show bato- and hypochromic changes upon increasing CT-DNA concentration.

Electronic absorption spectroscopy is universally employed to determine the possible binding modes of small molecules with DNA. The interaction of such molecules with DNA is characterized through absorption spectral titration, followed by the measurements of the observed hyperchromism or hypochromism and shift of the maximum absorption bands due to successive additions of DNA to a fixed concentration of complex [69]. The absorption spectra of **L** and **[Ag_2_L_2_]^2+^** in the absence and presence of CT-DNA, are given in Figure 3.

In general, the changes in the subsequent titration spectra are related to the changes occurring in the conformation and structure of the DNA helix. One may assume that the hypochromism arises from the changes of the DNA in the helix axis [70]. Moreover, a bathochromic shift, together with hypochromism, occurs due to the stacking effects between the base pairs and the aromatic chromophores [71]. Such an effect is observed for both studied compounds (Figure 3). Ligand **L** comprises the thiazole moieties which are able to interact with the DNA through π–π interactions and van der Waals forces. In complex, the thiazole and imine N coordinate the Ag(I) ions in the equatorial positions, thus giving flat, positively charged moieties with potential, unoccupied coordination sites in axial positions. In order to further evaluate and compare the binding affinity of ligand **L** and complex **[Ag_2_L_2_]^2+^**, the intrinsic binding constants K_b_ were determined by using the BindFit 0.5v module for supramolecular chemistry research and analysis. It revealed that the complex **[Ag_2_L_2_]^2+^** has a slightly higher affinity towards DNA helix than the ligand **L**, what is evidenced by the K_b_ = 5.94 × 10^6^ M^−1^ and K_b_ = 3.65 × 10^6^ M^−1^, respectively (Figure S6). These values correspond well to the literature data for other Ag(I) complexes [14,72,73]. In order to obtain more insights into the binding mechanism of the studied small molecules with DNA, the competitive binding studies with selective fluorescent dyes were performed and are discussed in the following section.

#### 3.3.2. Competitive Studies with Fluorescent Dyes

Two fluorescent dyes were chosen for this study—ethidium bromide (EB) and Hoechst 33258. Both dyes are weak luminescent compounds exhibiting high affinity to certain regions in the DNA helix and their emission is enhanced after formation of the dye–DNA complexes. EB is reported as an intercalator and Hoechst 33258 as minor groove binder. In general, introduction of a competing compound may displace EB and Hoechst 33258 directly or indirectly (through conformational changes resulting in the dyes ejection), thus providing valuable information regarding the possible binding modes [74].

**Figure 4:**
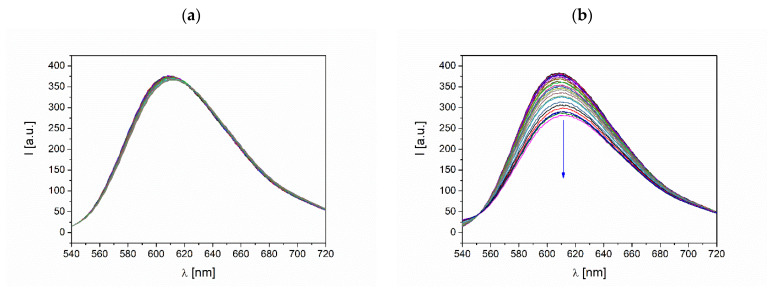
Emission spectra of EB bound to CT-DNA in the presence of increasing concentrations of (**a**) **L** and (**b**) **[Ag_2_L_2_]^2+^** (from 0 to 190 μM); ([Ethidium Bromide] = 20 μM, [CT-DNA] = 26 μM). Arrow shows the hypochromic changes upon increasing concentration of **[Ag_2_L_2_]^2^**^+^.

Displacement of EB from its EB–DNA complex due to the gradual addition of competing molecule results in subsequent quenching of emission bands. However, if the diminution of emission is lower than 50% it can be concluded that, in fact, the compounds do not competitively intercalate the DNA^13^. This may also occur to some extent when groove binding or other electrostatic interactions occur in the system. Such a phenomenon was observed in case of **[Ag_2_L_2_]^2+^**, otherwise than for ligand **L** which did not expel EB at all (as no diminution of emission appeared; Figure 4).

**Figure 5:**
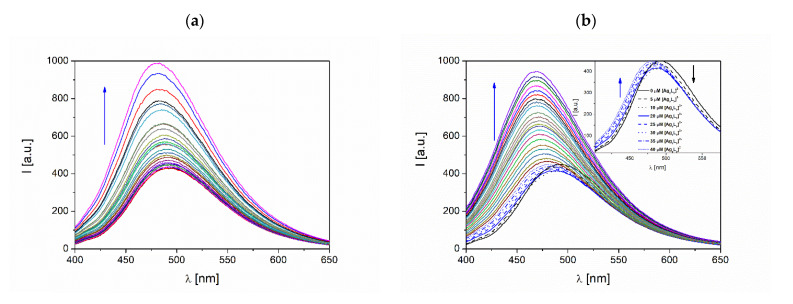
Emission spectra of Hoechst 33258 bound to CT-DNA in the presence of increasing concentrations of (**a**) **L** and (**b**) **[Ag_2_L_2_]^2+^** (from 0 to 150 μM); ([Hoechst 33258] = 10 μM, [CT-DNA] = 20 μM). Arrows show the hyperchromic changes upon increasing concentrations of compounds.

Typically, when the competing minor groove binder is added to the Hoechst 33258-DNA complex the emission is quenched due to the displacement of the dye [75]. However, in this case the behavior of the tested compounds is of particular interest, since they do not decrease the emission but enhance it (Figure 5). The significant hyperchromic effect accompanied by the hypsochromic shift of the emission maximum indicate the existence of strong interaction between the Hoechst 33258-DNA complex and the tested compounds, which does not involve dye displacement. A possible interaction in this case involves the external electrostatic interactions between the compounds and Hoechst 33258-DNA system. Similarly, the fluorescence of DAPI, another minor groove binder, was enhanced in its complex with DNA which was attributed to its binding with the derivatives of barbituric acids including Ag(I) complex [76]. As depicted in Figure 5, the binding behavior between **L** and **[Ag_2_L_2_]^2+^**, however, slightly differ. The addition of equimolar amount of **[Ag_2_L_2_]^2+^** (10 μM) to the Hoechst 33258-DNA complex results in a slight decrease in the fluorescence, suggesting partial dye displacement followed by intense enhancement of the emission, which indicates external binding.

#### 3.3.3. DNA Melting Studies

**Figure 6:**
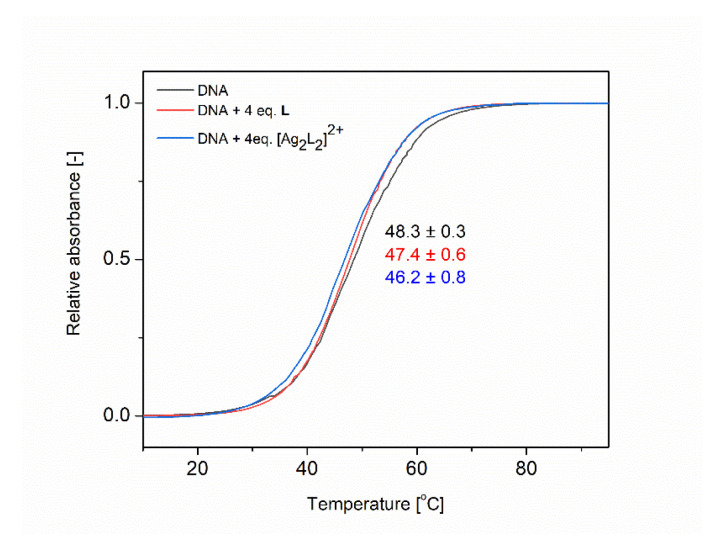
UV thermal melting curves of d(GTTAATCGCTGG) DNA alone (2.5 μM) and together with ligand **L** and complex **[Ag_2_L_2_]^2+^** (10 μM).

A DNA melting experiment allows one to define structural changes in nucleic acids, as well as to determine the stability of a secondary DNA structure [77]. In our study, complex **[Ag_2_L_2_]^2+^** (T_m_ = 46.2 ± 0.8 °C) and ligand **L** (T_m_ = 47.4 ± 0.6 °C) caused a decrease in the T_m_ of DNA (T_m_ = 48.3 ± 0.3 °C), suggesting that destabilizing interactions of the compounds with the 12-oligomer occurred (Figure 6). It is especially visible in the case of complex **[Ag_2_L_2_]^2+^** that led to a destabilization of about 2 °C. It may explain the ability of this compound to partially push out the EB and Hoechst-33258 from their luminescent complexes with DNA (*cf.* Section 3.3.1 and Section 3.3.2) due to the external electrostatic binding leading to significant structural perturbations. Such structural changes may result in the replication impairment causing the cell apoptosis.

### 3.4. Binding of BSA—A Model Protein

Serum albumin is a protein that mainly plays a transportation role for many compounds, such as hormones and fatty acids, in vertebrates. In general, the weak binding is a cause of a poor distribution, while strong binding significantly decreases the concentration of a drug in blood plasma [78]. An understanding of the mechanism by which metallodrugs bind to and transport through the body is crucial for the development of effective drugs. Moreover, serum albumins are found to accumulate in the tumor and inflammatory cells, which makes them a valuable target for these therapies [79].

Bovine Serum Albumin (BSA) is a model protein used in the studies of interaction with various compounds due to its high homology (76%) to Human Serum Albumin (HSA). In order to evaluate the ability of the compounds to bind to BSA and to shed light on the mechanism of these interactions, various spectroscopic methods were used, such as: CD, fluorescence and synchronous fluorescence [80,81].

#### 3.4.1. Conformational Changes Detected by CD

**Figure 7:**
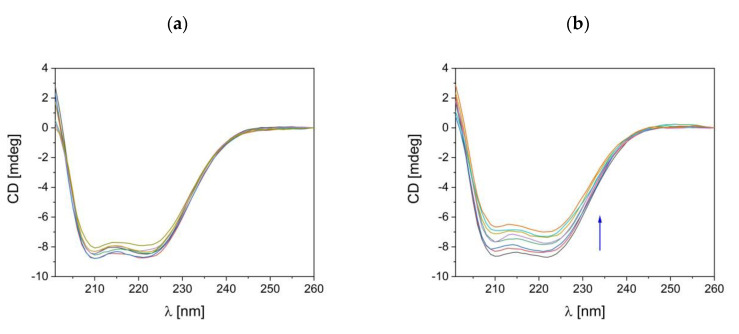
CD spectra of BSA (75 nM) titrated with (**a**) **L** (0-350 equiv.) and (**b**) **[Ag_2_L_2_]^2+^** (0–350 equiv.) in PBS (pH = 7.4).

Circular dichroism is a very sensitive method for determining changes in the secondary structure of proteins. BSA possess two characteristic peaks at 208 and 222 nm [82], which are associated with the π–π^⁎^ and n–π^⁎^ transitions for the α-helix peptide bond [83]. In our studies the content of α-helix decreased ca. 16% suggesting that **[Ag_2_L_2_]^2+^**, indeed, bound to BSA, causing some structural changes in its secondary structure (Figure 7). Ligand **L**, however, did not cause significant structural perturbations of the tested serum albumin. Liu et al., in their work, reported that scoparone, a biological active substance derived from the traditional Chinese herbal medicine, is transported by serum albumins through binding with them, which is evidenced by the decrease in α-helix content [84].

#### 3.4.2. Fluorescence Quenching Studies

The interactions of **L** and **[Ag_2_L_2_]^2+^** with BSA were further evaluated by the quenching of the inner fluorescence of BSA with increasing amounts of tested compounds. Fluorescence of BSA arises from the presence of Trp, Tyr and Phe aminoacids [85], among which the residues Trp-134 and Trp-213 are mainly responsible for it [86].

**Figure 8:**
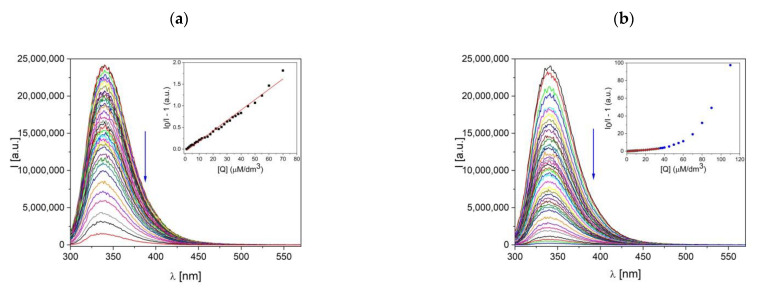
Fluorescence spectra of BSA (5 μM) in the presence of increasing concentrations of (**a**) **L** (0–170 μM) and (**b**) **[Ag_2_L_2_]^2+^** (0–110 μM). Inset: Stern–Volmer plot of I_0_/I−1 vs. [**L**] or [**[Ag_2_L_2_]^2+^**] for the titration of BSA; ▪, experimental data points; solid line, linear fitting of the data.

As seen in Figure 8, both compounds quench the emission of BSA, indicating binding of both compounds to the protein. On the basis of the obtained numerical data, the constants of Stern–Volmer (Equation (3)) were calculated: 8.80 × 10^4^ for complex (R^2^ = 0.98; 0–35 μM) and 2.40 × 10^4^ for ligand (R^2^ = 0.99; 0–70 μM). As the titration of ligand **L** gives a linear plot dependence (inset in Figure 8a) suggesting only one type of interaction with protein (either static or dynamic), then in case of **[Ag_2_L_2_]^2+^** this dependce is linear only between 0 and 70 μM and above starts to grow expotentially (inset in Figure 8b), thus suggesting the mixed type of interactions [87].

#### 3.4.3. Binding Parameters

**Figure 9:**
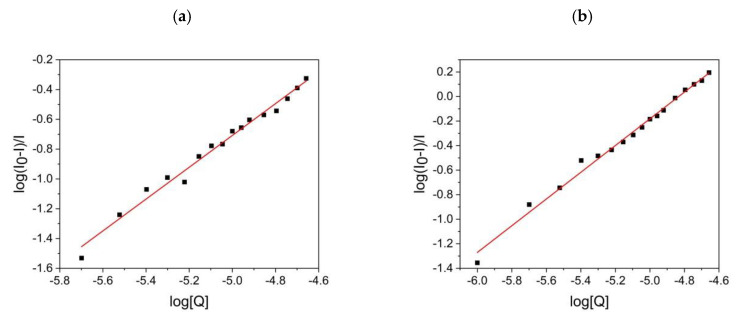
Scatchard plot of fluorescence titration of BSA (5μM) with (**a**) **L** (0–22 μM) and (**b**) **[Ag_2_L_2_]^2+^** (0–22 μM).

The binding constant K_bin_ allows us to determine whether a given compound binds to the protein strongly enough to be delivered to the target site and not too strongly to be released. Another important parameter is the number of binding sites, n, which informs about the number of target sites on albumin for the compound. These parameters can be determined from the Scatchard equation (Equation (4)) and are presented in Table 2 and Figure 9 **[88]**. The n values are close to one, suggesting that both **L** and **[Ag_2_L_2_]^2+^** bind the BSA in molar ratio 1:1. In such a case when small molecules bind to the protein independently and in a constant ratio, the comparison of the K_bin_ constant provides valuable information on the affinity of molecules to the biomacromolecule. In our studies, a preference of BSA to bind with **[Ag_2_L_2_]^2+^** is ca. 4 times higher (K_bin_ = 1.84 × 10^5^ M^−1^) than to **L** (K_bin_ = 4.27 × 10^4^ M^−1^). These moderate K_bin_ values give good perspective for the efficient transportation of the tested compounds in the blood plasma and release in the target cells.

#### 3.4.4. Synchronous Fluorescence Spectroscopic Studies of BSA

**Figure 10:**
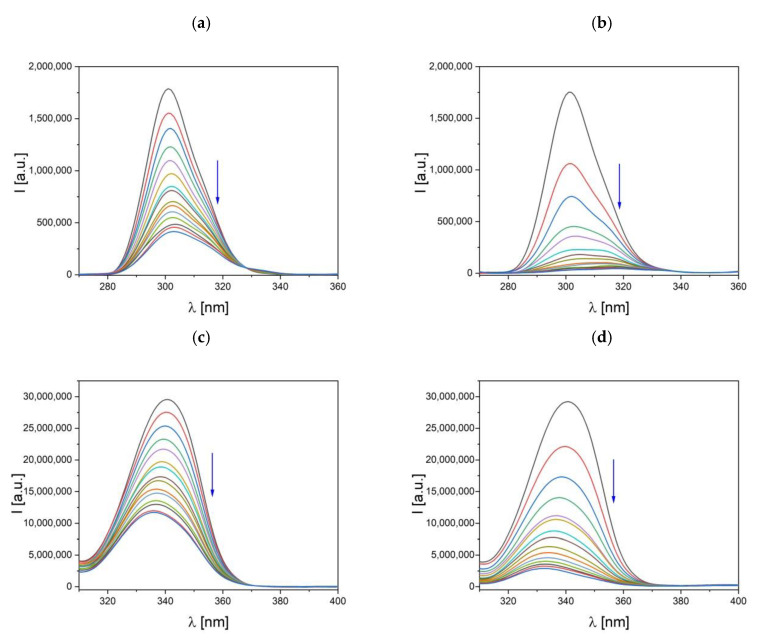
Changes in synchronous fluorescence spectra of BSA (5 μM) at Δλ = 15 nm (Tyr) upon titration by (**a**) **L** and (**b**) **[Ag_2_L_2_]^2+^** and at Δλ= 60 nm (Trp) upon titration by (**c**) **L** and (**d**) **[Ag_2_L_2_]^2+^**.

Since the introduction of synchronous fluorescence in 1970 by Lloyd and Evett [89] it became possible to determine whether the molecular microenvironment around the chromophore is changing. The tracked chromophores of BSA are Tyr and Trp moieties–Δλ = 15 nm is characteristic for the Tyr, while Δλ = 60 nm for Trp residues [90,91]. The maximum emission wavelengths of Trp and Tyr residues in BSA are related to the polarity of their surroundings; therefore any changes occurring in the emission maxima reflect conformational changes in the close vicinity of these moieties. Figure 10 shows the effects of **L** and **[Ag_2_L_2_]^2+^** on the spectra of Tyr and Trp. In both cases, reduction in emission was observed. However, the emission maxima blueshift 5 and 9 nm in presence of **L** and **[Ag_2_L_2_**]^2+^, respectively, only when Δλ is equal to 60 nm (Figure 10c,d). This indicates changes in the microenvironment related to hydrophobicity in close vicinity of the Trp residue [84]. On contrary, the microenvironment around the Tyr moiety did not change, as no shifts in fluorescence maxima were observed (Figure 10a,b).

## 4. Conclusions

The possible binding mechanisms to relevant biomolecules, DNA and BSA, of the new Schiff base ligand **L** and its bimetallic complex **[Ag_2_L_2_](PF_6_)_2_** were investigated by spectroscopic methods. The complex **[Ag_2_L_2_](PF_6_)_2_** partially pushes out the EB and Hoechst-33258 from their luminescent complexes with DNA due to the external electrostatic binding leading to significant structural perturbations of the helix. Such structural changes may result in the replication impairment causing cell apoptosis. The binding constants K_bin_ obtained for the studied compounds with BSA are in the intermediate range so that they are not too low to prevent efficient distribution and are not so high to lead to decreased plasma concentration. Both compounds bind in similar regions of the protein, therefore one can assume that the binding site is dictated by the ligand, but the strength of the interaction is increased by the complexed metal ions.

The biological significance of this work is evident since albumins serve as carriers of drugs to inflammatory and cancerous cells through blood plasma [86]. It was also reported previously [11] that Ag(I) complexes can accumulate in the nucleus and cause the cell’s death as a result of binding with DNA. Therefore, the Ag(I) based metallodrugs binding with albumins with the intermediate strength and interacting with DNA are valuable candidates for future therapies.

## Figures and Tables

**Table 1:** Selected geometrical data (Å, °) with s.u.’s in parentheses; ^i^ denotes symmetry operation 1-x,2-y,1-z.

	L	[Ag_2_L_2_]^2+^
Ag1-N4		2.144(3)
Ag1-N14^i^		2.192(3)
Ag1-N20^i^		2.479(3)
N4-Ag1-N14^i^		166.20(11)
N4-Ag1-N20^i^		120.12(10)
N14^i^-Ag1-N20^i^		73.34(10)
C2-S1-C5	89.32(5)	89.82(18)
C3-N4-C5	109.44(18)	111.1(3)
C16-S17-C18		89.30(18)
C16-N20-C19		110.3(3)

**Table 2:** Number of binding sites (n) and binding constant (K_bin_) for complex and ligand with BSA.

Compound	N	K_bin_ [M^−1^]
**L**	1.06	4.27 × 10^4^
**[Ag_2_L_2_]^2+^**	1.08	1.84 × 10^5^

## Data Availability

All data availability is in supplementary data and any other data will be with corresponding author. Bindfit data are available at http://app.supramolecular.org/bindfit/view/Fit (accessed on 21 September 2021): http://app.supramolecular.org/bindfit/view/a89dc1ed-1796-4f2a-92ca-99ccd419a915 for ligand **L** and http://app.supramolecular.org/bindfit/view/73fad0f0-bab1-4c1c-8990-5fddd7fb9b34 for complex **[Ag_2_L_2_]^2+^**.

## Supplementary Materials

The following are available online at https://www.mdpi.com/article/10.3390/biom11101449/s1, Figure S1: IR spectra of **L** and **[Ag_2_L_2_]^2+^** in KBr. Figure S2: 13C NMR spectra for ligand **L** measured in (CD3)2SO; Figure S3: Stability tests of **L** (a) and **[Ag_2_L_2_]^2+^** (b) (20 µM) in PBS buffer (pH = 7.4) with 1% MeCN content. The tests were carried out from 0 to 120 min; Figure S4: Stability tests of **L** (a) and **[Ag_2_L_2_]^2+^** (b) (20 μM) in Tris-HCl buffer (pH = 7.2) with 1% MeCN content. The tests were carried out from 0 to 120 min; Figure S5: A crystal packing as seen along x-direction. The anions and solvent molecules are shown in the van der Waals radii representation. Figure S6: Bindfit fitting of UV-Vis titrations of **L** (**a**) and **[Ag_2_L_2_]^2+^** (**b**) generated using website supramolecular.org.
