# Supplementary material for: Synthesis and Spectroscopic Investigations of Schiff Base Ligand and Its Bimetallic Ag(I) Complex as DNA and BSA Binders
Source: Biomolecules. 2021 Oct 2;11(10):1449. doi: 10.3390/biom11101449 (PMC8533391; doi:10.3390/biom11101449)
Supplement: Supplementary file 1 [file biomolecules-11-01449-s001.zip › biomolecules-1383047-supplementary.pdf]

biomolecules

MDPI

Electronic Supplementary Information

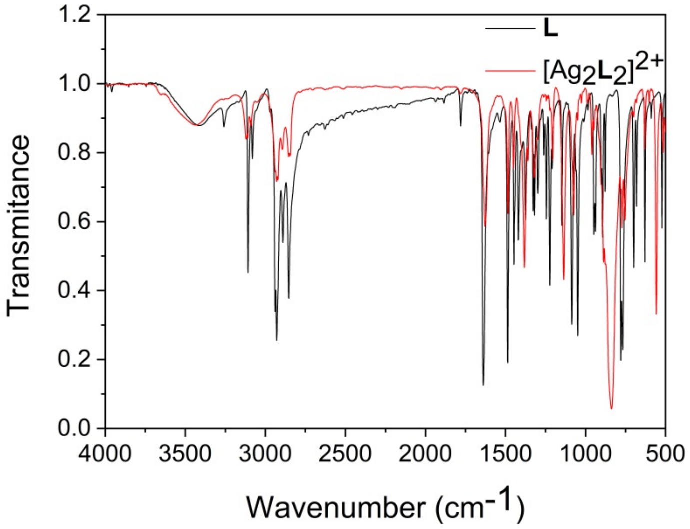

Figure S1. IR spectra of **L** and **[Ag_2L_2]$^{2+}$** in KBr.

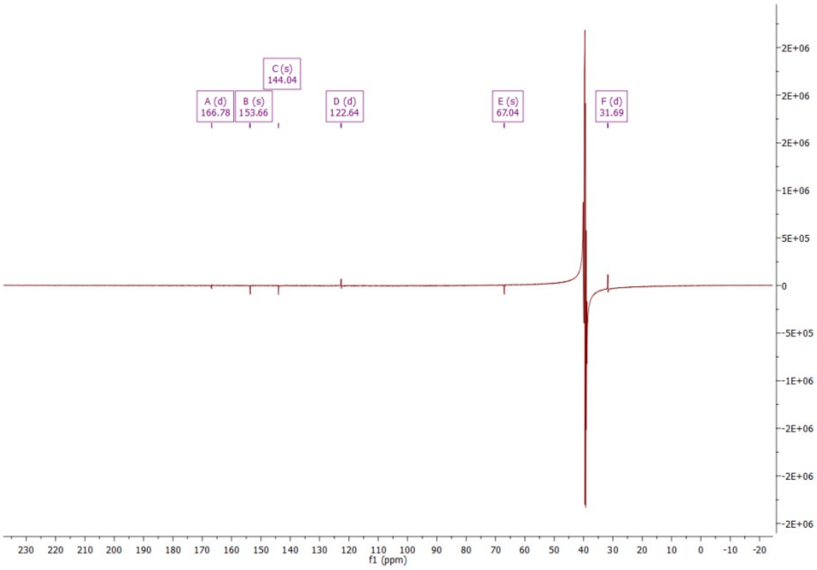

Figure S2. ^{13}C NMR spectra for ligand **L** measured in $(CD_3)_2SO$.

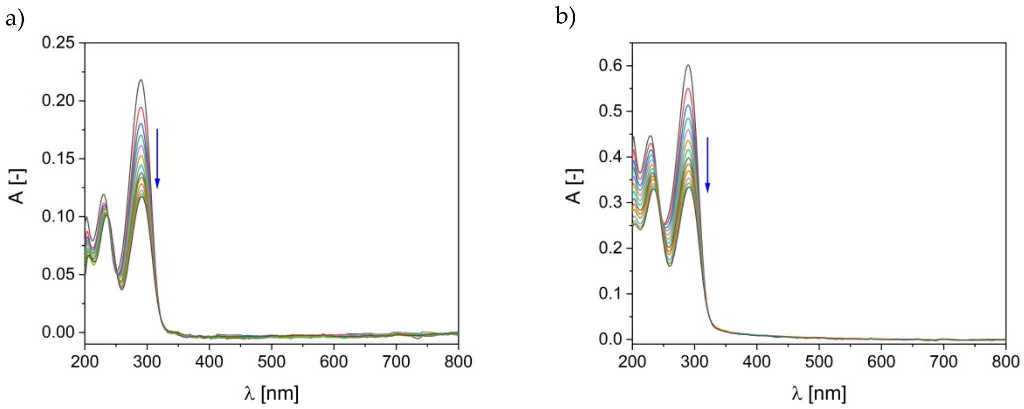

Figure S3. Stability tests of **L** (**a**) and **[Ag_2L_2]$^{2+}$** (**b**) (20 μM) in PBS buffer (pH = 7.4) with 1% MeCN content. The tests were carried out from 0 to 120 minutes.

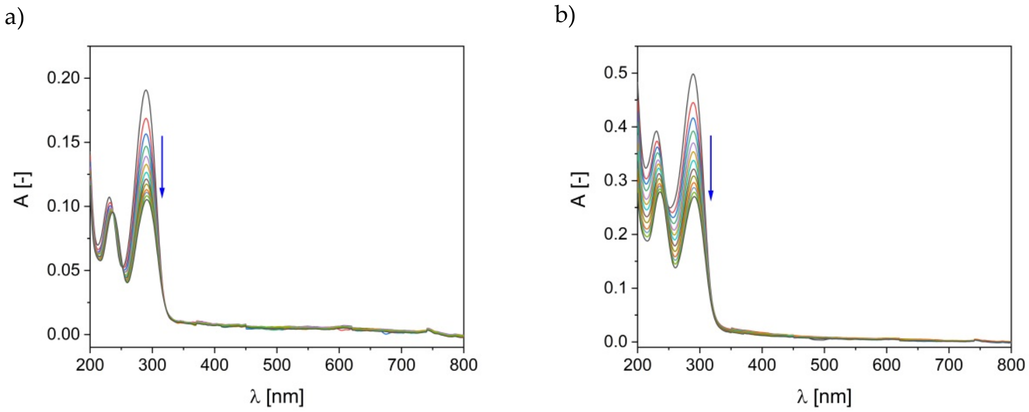

Figure S4. Stability tests of **L** (**a**) and **[Ag_2L_2]$^{2+}$** (**b**) (20 μM) in Tris-HCl buffer (pH = 7.2) with 1% MeCN content. The tests were carried out from 0 to 120 minutes.

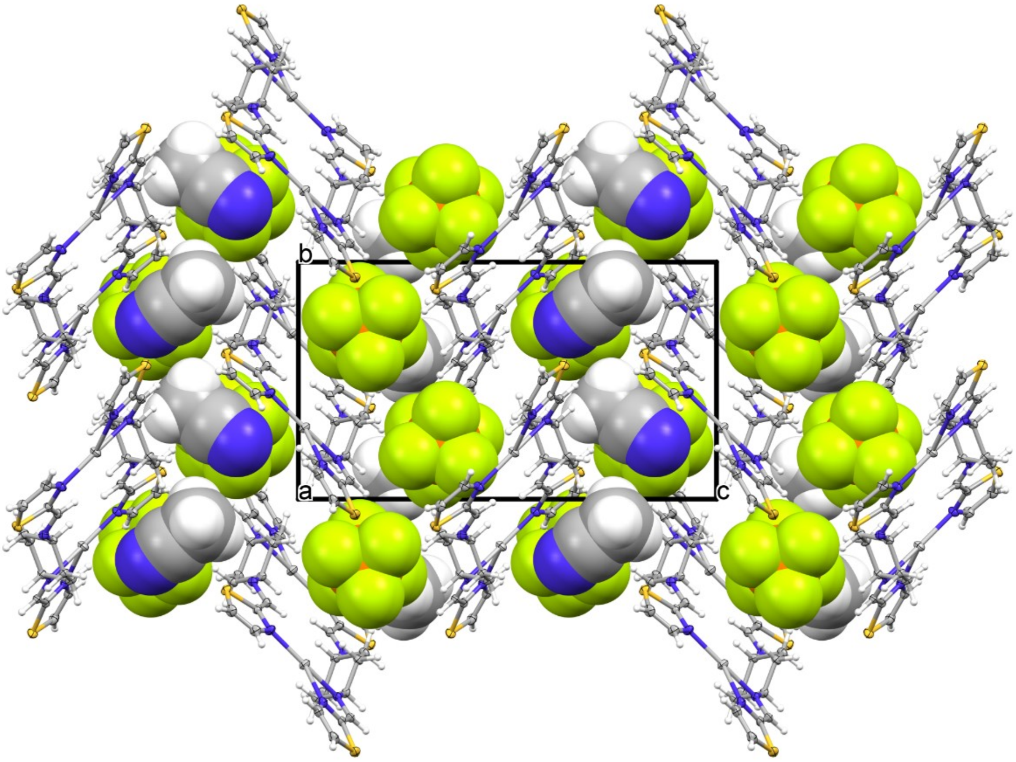

Figure S5. A crystal packing as seen along x-direction. The anions and solvent molecules are shown in the van der Waals radii representation.

a)

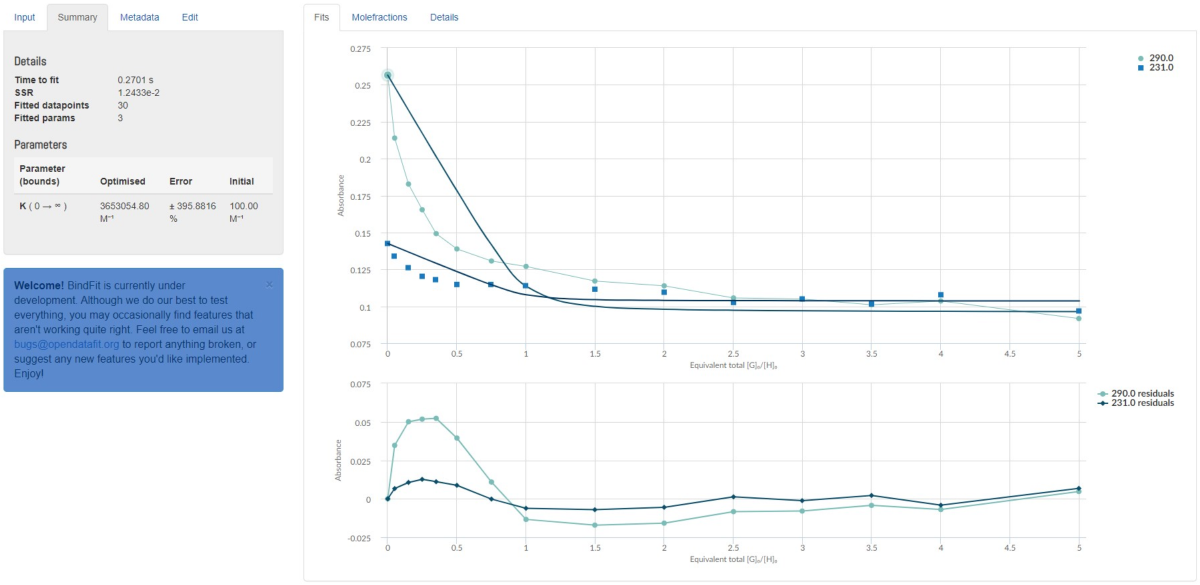

b)

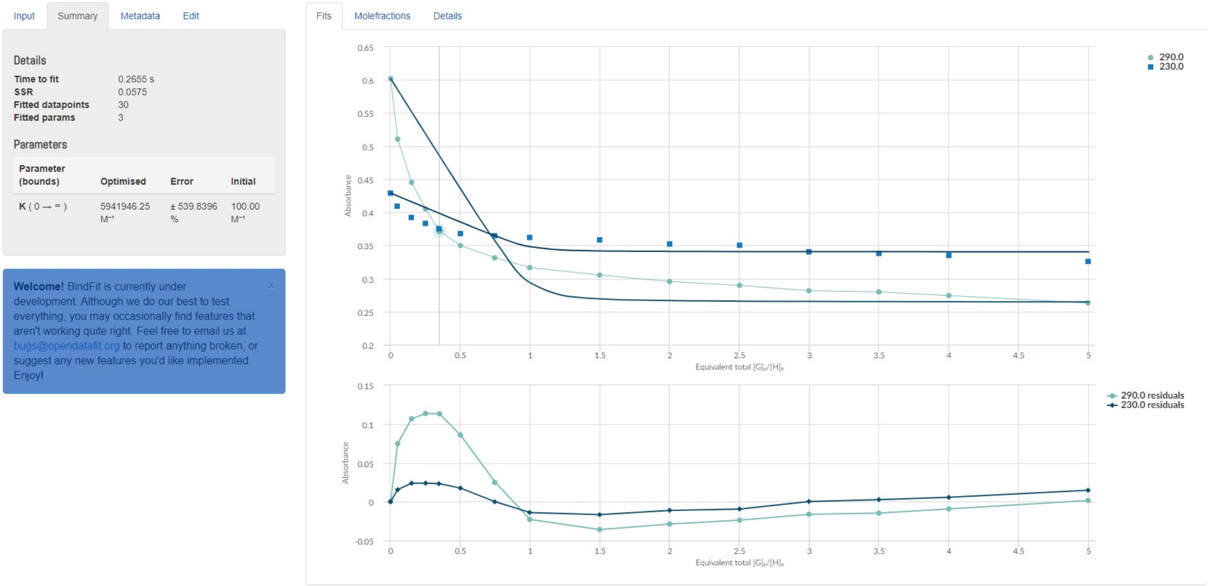

Figure S6. Bindfit fitting of UV-Vis titrations of **L** (**a**) and **[Ag_2L_2]$^{2+}$** (**b**) generated using website supramolecular.org.
